# Italy and COVID-19: the changing patient flow in an orthopedic trauma center emergency department

**DOI:** 10.1186/s13018-020-01816-1

**Published:** 2020-08-14

**Authors:** Francesco Luceri, Ilaria Morelli, Riccardo Accetta, Laura Mangiavini, Nicola Maffulli, Giuseppe M. Peretti

**Affiliations:** 1 IRCCS Istituto Ortopedico Galeazzi, Milan, Italy; 2 Residency Program in Orthopaedics and Traumatology, University of Milan, Milan, Italy; 3 Department of Biomedical Sciences for Health, University of Milan, Milan, Italy; 4 Department of Medicine, Surgery and Dentistry, University of Salerno, Via S. Allende, 84081 Baronissi, SA Italy; 5 School of Pharmacy and Bioengineering, Keele University School of Medicine, Thornburrow Drive, Stoke on Trent, England; 6 Centre for Sports and Exercise Medicine, Barts and The London School of Medicine and Dentistry, Queen Mary University of London, Mile End Hospital, 275 Bancroft Road, London, E1 4DG England

On 12 March 2020, the World Health Organization declared a pandemic by Coronavirus disease (COVID-19) [1]. Despite the lockdown measures adopted to stop the spread of SARS-CoV-2, we are dangerously close to 400,000 deaths worldwide [2].

In Northern Italy, the overwhelming number of COVID-19 patients required a complete reorganization of the healthcare system [3, 4]: wards were converted into COVID-19 care units, and deferrable surgeries and outpatient consultations were suspended. Some hospitals were designated hubs for specific urgent conditions [5], with the need to maximize resources and reduce patient crowding, reducing potential nosocomial COVID-19 spread.

Major changes in the patient flow at the emergency department (ED) of Galeazzi Orthopaedic Institute in Milan, a major trauma center, were evident. The analysis of this aspect during the first month of the pandemic (12 March to 12 April 2020) compared to the same period in 2019 demonstrated marked differences in length of emergency department stay, request for chest radiographs, discharge diagnosis, triage color-code at admission and discharge (*white code*: non-urgent patients; *green code*: urgent but non-critical patients; *yellow code*: fairly critical patients; *red code*: patients at danger of death), and emergency department arrival and discharge modalities.

The number of patients in this 1-month period was 2558 in 2019 and 670 in 2020, an overall patient flow reduction of 73.8%. Patients’ demographics and diagnoses at discharge are summarized in Table 1. Average patients’ age was significantly higher in 2020 than in 2019 (*t*_(3226)_ = 14.75, *p* < 0.0001), with a marked reduction in the number of pediatric emergencies (age ≤ 18 years old) during lockdown (OR 0.31, 95% CI 0.23–0.42, *p* < 0.0001). The mean emergency department length of stay significantly decreased in 2020 (*t*_(3226)_ = 10.85, *p* < 0.0001). Furthermore, during the pandemic, more chest plain radiographs were requested (OR 6.11, 95% CI 4.81–7.77, *p* < 0.0001). The number of patients discharged with a diagnosis other than “fracture” (therefore including sprains, contusions, back pain) was markedly reduced (OR 0.24, 95% CI 0.20–0.28, *p* < 0.0001) in 2020. On the other hand, both proximal femoral fractures showed a remarkable increase (OR 13.6, 95% CI 9.31–19.85, *p* < 0.0001) during the pandemic, as did the overall rate of fragility fractures in the elderly (OR 7.57, 95% CI 5.87–9.76, *p* < 0.0001).

**Table 1:** Demographics and clinical data in standard and pandemic conditions

		NG	PG	Absolute variation	Relative variation
**Sex:** ***N*** **(%)**	**Male**	1323 (51.7)	297 (44.3)	***− 77.5%***	***− 7.4%***
	**Female**	1235 (48.3)	373 (55.7)	***− 69.5%***	***7.4%***
**Age: mean ± SD (years)**		41.2 ± 23.5 [0, 98]	56.3 ± 23.9 [0, 99]	***36.7%***	***-***
**ED stay ± SD (min)**		146 ± 63 [2, 839]	106 ± 140 [5, 180]	***− 27.4%***	***-***
**Pediatric patients (%)**		633 (24.7)	62 (9.2)	***− 90.2%***	***− 15.5%***
**Chest radiographs (%)**		145 (5.7)	180 (26.9)	***24.1%***	***21.2%***
**Diagnosis (%)**	***Clavicle fractures***	13 (0.5)	5 (0.7)	***− 61.5%***	***0.2%***
	***Proximal humeral fractures***	30 (1.2)	25 (3.7)	***− 16.7%***	***2.5%***
	***Humeral shaft fractures***	2 (0.1)	8 (1.2)	***300%***	***1.1%***
	***Elbow fractures***	45 (1.7)	14 (2.1)	***− 68.9%***	***0.4%***
	***Wrist and hand fractures***	202 (7.9)	73 (10.9)	***− 63.9%***	***3%***
	***Vertebral fractures***	15 (0.6)	6 (0.9)	***− 60%***	***0.3%***
	***Proximal femoral fractures***	38 (1.5)	114 (17.0)	***200%***	***15.5%***
	***Other femoral fractures***	6 (0.2)	9 (1.4)	***50%***	***1.2%***
	***Patellar fractures***	15 (0.6)	5 (0.7)	***− 66.7%***	***0.1%***
	***Tibia fractures***	27 (1.1)	12 (1.8)	***− 44.4%***	***0.7%***
	***Foot and ankle fractures***	161 (6.3)	59 (8.8)	***− 63.4%***	***2.5%***
	***Pneumonia***	3 (0.1)	14 (2.1)	***366.7%***	***2%***
	***Other***	2001 (78.2)	336 (48.7)	***− 83.2%***	***− 29.5%***
**Total**		**2558**	**670**	***− 73.8%***	

*SD* standard deviation, *NG* non-pandemic group (2019), *PG* pandemic group (2020)

Table 2 reports the triage codes at admission and discharge. A reduction of 8.9% and 14.1% for white and green codes, respectively, was found in the pandemic month. As expected, comparing the walking wounded (green and white codes) and urgent patient (yellow and red codes) rates in 2019 and 2020, an odds ratio of 0.12 (95% CI 0.09–0.15, *p* < 0.0001) was found. Similarly, triage at discharge presented a reduction of the white codes and a relative increase of all the other triage categories (OR 0.56, 95% CI 0.44–0.70, *p* < 0.0001). 
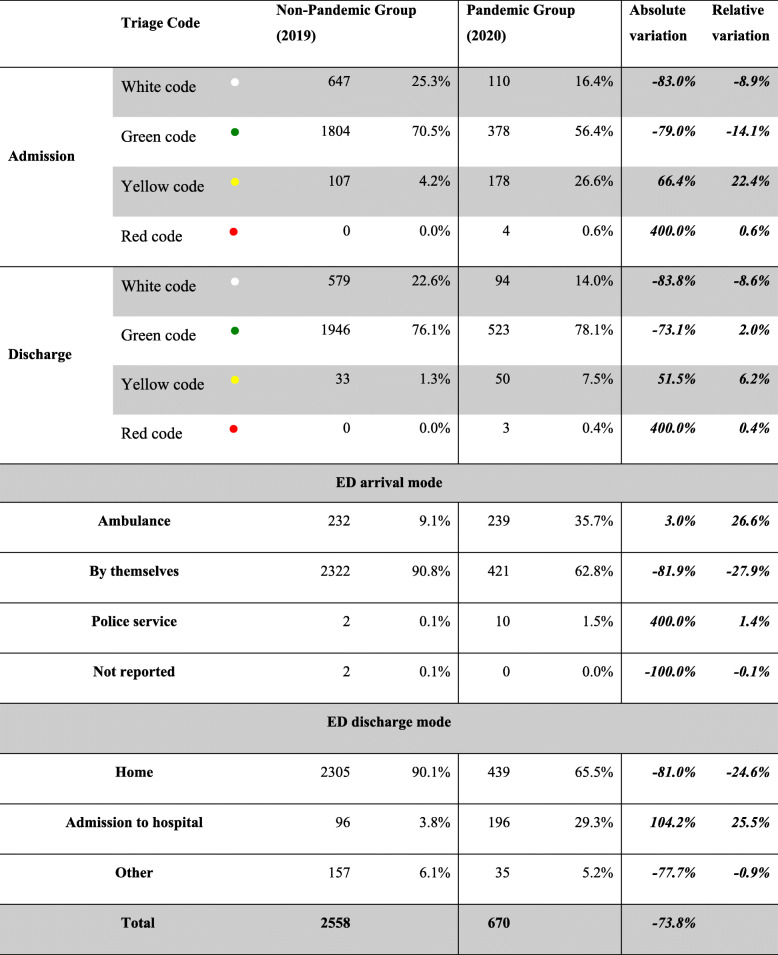

**Table 2:**
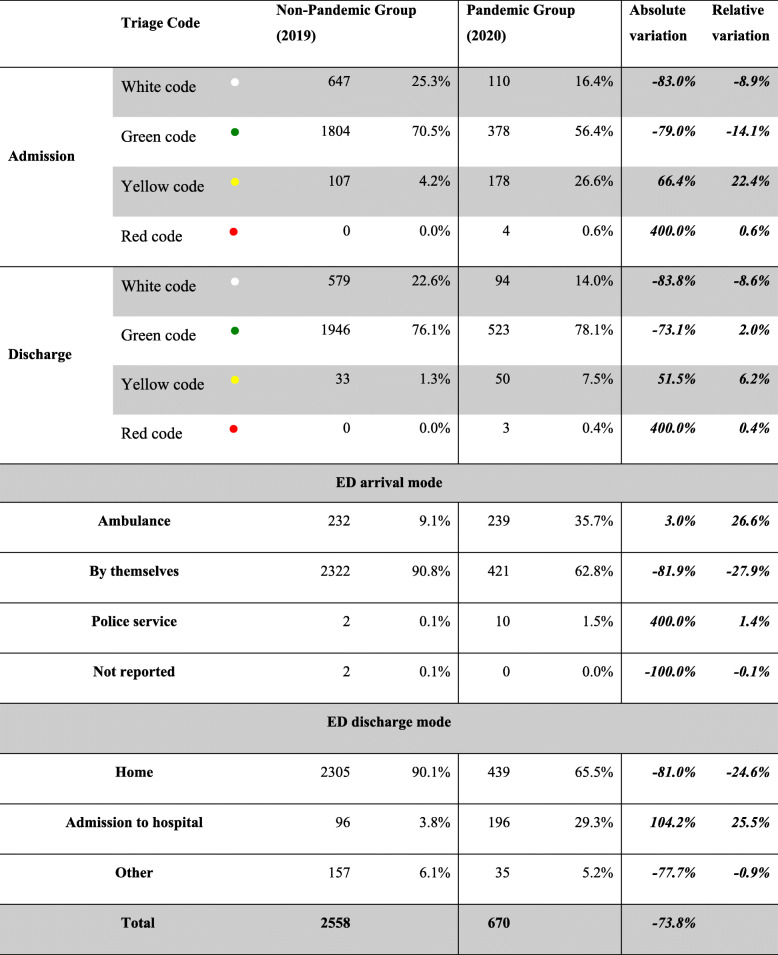
Priority categories (triage code) at admission and discharge in standard and pandemic conditions

Emergency department arrival mode and emergency department discharge mode in the two conditions

*ED* emergency department

The rate of patients brought to emergency department by ambulance increased in 2020 (Table 2) (OR 5.56, 95% CI 4.52–6.84, *p* < 0.0001). Finally, in 2020 more patients were hospitalized and fewer were discharged home (OR 10.72, 95% CI 8.23–13.97, *p* < 0.0001).

COVID-19 produced not only an overcrowding of healthcare facilities by patients with severe respiratory syndromes, but also a change in emergency department patient flow. There was a marked reduction in the number of pediatric emergency in this pandemic period and an increased proportion of proximal femoral fractures. The pattern of emergency department outflow also changed, with a significant reduction of the emergency department stay, an increase of 25% in patients requiring urgent hospital admission, and a decrease of 24% in patients discharged at home.

Since 10 March 2020, the Italian populace have been allowed to leave home only for proved and undefeatable reasons such as work (i.e., healthcare professionals), buying food or essential goods, and urgent health reasons. Restriction of social contacts, open-air activities, and sports performed in gyms and swimming pools were deemed the safest measure in the absence of a vaccine and efficient medical therapies [6–8]. These measures led to a drop of patients presenting for non-urgent chronic reasons (such as tendinopathy, back pain, osteoarthritis-related pain), sports-related injuries (sprains, contusions, dislocations, minor fractures), and minor road accidents. Therefore, fewer minor traumas came to emergency department, explaining the decreasing percentages of non-urgent admission codes, autonomous emergency department arrivals, and home discharge. For the same reasons, pediatric emergencies decreased by 15.5%. On the other hand, trauma was concentrated in regional hubs, leading to a greater number of patients with severe trauma requiring surgery: fragility fractures, such as those involving proximal femur especially, and humerus, vertebral, and pelvic branch fractures in older adults, increased in percentage during lockdown.

The COVID-19 pandemic has led to an increase in the number of required chest plain radiography, usually needed only in case of rib fractures or for patients over 45 years before surgical treatment. Patients admitted for COVID-19 share with femoral fractures common features such as the arrival by ambulance, the yellow code at admission, and the rapid admission to hospital. Nevertheless, the increase in yellow code patients was only 6.2% compared to 2019.

The first month of COVID-19 pandemic led to a 73% reduction in the overall emergency department patient flow of our Regional Trauma Hub, specifically set up in response to this worldwide disaster. A similar decrease in emergency department patient flow was reported in Canada, Taiwan, and Hong Kong during the SARS epidemic (2003–2004), and this should be partially attributed to people’s perception of the emergency department as a place of infection [9–11].

In this COVID-19 era, frontline medical staff in emergency departments are facing new challenges to diagnose and treat patients [12]. Understanding the trend of patient flow in a trauma hub emergency department is important to better manage the preventive isolation of each patient attending this service. A key strategic element is demand forecast to help staff to plan their activities in the long and the short-term [13]. The effects of the worldwide pandemic on several surgical activities have been scantily reported [14–16]. For what concerns emergency department, reports have been focusing only on targeted SARS-CoV-2 test programs [17–19].

Social isolation certainly reduced the risk of trauma among the general population, and the fear of contagion probably kept non-urgent patients away from the emergency department. Evidence-based programs are fundamental to identify new strategies to maximize National Health System resources and decrease the time which patients spend in the emergency department, reducing overcrowding.

## Data Availability

Institutional Review Board Approval.

## References

[1] World Health Organization (2020). WHO director-general’s opening remarks at the media briefing on COVID-19 - 11 March 2020.

[2] World Health Organization (2020). Coronavirus disease 2019 (COVID-19) situation report – 130.

[3] Livingston E, Bucher K. Coronavirus disease 2019 (COVID-19) in Italy. JAMA. 2020. 10.1001/jama.2020.4344. 10.1001/jama.2020.4344 32181795

[4] Cipollaro L, Giordano L, Padulo J (2020). Musculoskeletal symptoms in SARS-CoV-2 (COVID-19) patients. J Orthop Surg Res.

[5] Chisci E, Masciello F, Michelagnoli S. Creation of a vascular surgical Hub responding to the COVID-19 emergency: the Italian USL Toscana Centro model. J Vasc Surg. 2020. 10.1016/j.jvs.2020.04.019. 10.1016/j.jvs.2020.04.019 PMC7160640 32305384

[6] Patel A, Jernigan DB, Abdirizak F (2020). Initial public health response and interim clinical guidance for the 2019 novel coronavirus outbreak - United States, December 31, 2019-February 4, 2020. Morb Mortal Wkly Rep.

[7] Tobías A. Evaluation of the lockdowns for the SARS-CoV-2 epidemic in Italy and Spain after one month follow up. Sci Total Environ. 2020;138539. 10.1016/j.scitotenv.2020.138539. 10.1016/j.scitotenv.2020.138539 PMC7195141 32304973

[8] De Girolamo L, Peretti GM, Maffulli N, et al. Covid-19-the real role of NSAIDs in Italy. J Orthop Surg Res. 2020;15. 10.1186/s13018-020-01682-x. 10.1186/s13018-020-01682-x PMC7197233 32366317

[9] Man CY, Yeung RSD, Chung JYM, et al. Impact of SARS on an emergency department in Hong Kong. Emerg Med. 2003. 10.1046/j.1442-2026.2003.00495.x. 10.1046/j.1442-2026.2003.00495.x 14992054

[10] Huang HH, Yen DHT, Kao WF, et al. Declining emergency department visits and costs during the severe acute respiratory syndrome (SARS) outbreak. J Formos Med Assoc. 2006. 10.1016/S0929-6646(09)60106-6. 10.1016/S0929-6646(09)60106-6 PMC7135596 16440068

[11] Heiber M, Lou WYW. Effect of the SARS outbreak on visits to a community hospital emergency department. Can J Emerg Med. 2006. 10.1017/S148180350001397X. 10.1017/s148180350001397x 17338843

[12] Yang Y, Yu A, Xiao W (2020). Strategies suggested for emergency diagnosis and treatment of traumatic orthopedics in the epidemic period of Corona Virus Disease 2019. Chinese J Orthop Trauma.

[13] Afilal M, Yalaoui F, Dugardin F, et al. Forecasting the emergency department patients flow. J Med Syst. 2016;40. 10.1007/s10916-016-0527-0. 10.1007/s10916-016-0527-0 27272135

[14] Di Saverio S, Pata F, Gallo G, et al. Coronavirus pandemic and colorectal surgery: practical advice based on the Italian experience. Color Dis. 2020. 10.1111/codi.15056. 10.1111/codi.15056 32233064

[15] Tuech J-J, Gangloff A, Di Fiore F, et al. Strategy for the practice of digestive and oncological surgery during the Covid-19 epidemic. J Visc Surg. 2020. 10.1016/j.jviscsurg.2020.03.008. 10.1016/j.jviscsurg.2020.03.008 PMC7269902 32249098

[16] Zou J, Yu H, Song D (2020). Advice on standardized diagnosis and treatment for spinal diseases during the coronavirus disease 2019 pandemic. Asian Spine J.

[17] Tolia VM, Chan TC, Castillo EM. Preliminary results of initial testing for Coronavirus (COVID-19) in the emergency department. West J Emerg Med. 2020;21. 10.5811/westjem.2020.3.47348. 10.5811/westjem.2020.3.47348 PMC7234708 32223871

[18] Huang Z, Zhao S, Li Z, et al. The battle against coronavirus disease 2019 (COVID-19): emergency management and infection control in a radiology department. J Am Coll Radiol. 2020. 10.1016/j.jacr.2020.03.011. 10.1016/j.jacr.2020.03.011 PMC7118524 32208140

[19] Lin M, Beliavsky A, Katz K (2020). What have we learned from patients investigated for COVID-19 so far? What can early Canadian experience screening for COVID-19 teach us about how to prepare for a pandemic?. CMAJ.

